# Perinatal Hypoxic-Ischemic Encephalopathy and Neuroprotective Peptide Therapies: A Case for Cationic Arginine-Rich Peptides (CARPs)

**DOI:** 10.3390/brainsci8080147

**Published:** 2018-08-07

**Authors:** Adam B. Edwards, Ryan S. Anderton, Neville W. Knuckey, Bruno P. Meloni

**Affiliations:** 1 Perron Institute for Neurological and Translational Science, Nedlands, WA 6009, Australia; ryan.anderton@nd.edu.au (R.S.A.); neville.knuckey@health.wa.gov.au (N.W.K.); bruno.meloni@perron.uwa.edu.au (B.P.M.); 2 School of Health Sciences and Institute for Health Research, The University of Notre Dame Australia, Fremantle, WA 6160, Australia; 3 Department of Neurosurgery, Sir Charles Gairdner Hospital, QEII Medical Centre, Nedlands, WA 6009, Australia; 4 Centre for Neuromuscular and Neurological Disorders, The University of Western Australia, Nedlands, WA 6009, Australia

**Keywords:** hypoxic-ischemic encephalopathy, hypoxia-ischemia, birth asphyxia, neuroprotection, cationic arginine-rich peptides, animal models

## Abstract

Perinatal hypoxic-ischemic encephalopathy (HIE) is the leading cause of mortality and morbidity in neonates, with survivors suffering significant neurological sequelae including cerebral palsy, epilepsy, intellectual disability and autism spectrum disorders. While hypothermia is used clinically to reduce neurological injury following HIE, it is only used for term infants (>36 weeks gestation) in tertiary hospitals and improves outcomes in only 30% of patients. For these reasons, a more effective and easily administrable pharmacological therapeutic agent, that can be used in combination with hypothermia or alone when hypothermia cannot be applied, is urgently needed to treat pre-term (≤36 weeks gestation) and term infants suffering HIE. Several recent studies have demonstrated that cationic arginine-rich peptides (CARPs), which include many cell-penetrating peptides [CPPs; e.g., transactivator of transcription (TAT) and poly-arginine-9 (R9; 9-mer of arginine)], possess intrinsic neuroprotective properties. For example, we have demonstrated that poly-arginine-18 (R18; 18-mer of arginine) and its D-enantiomer (R18D) are neuroprotective in vitro following neuronal excitotoxicity, and in vivo following perinatal hypoxia-ischemia (HI). In this paper, we review studies that have used CARPs and other peptides, including putative neuroprotective peptides fused to TAT, in animal models of perinatal HIE. We critically evaluate the evidence that supports our hypothesis that CARP neuroprotection is mediated by peptide arginine content and positive charge and that CARPs represent a novel potential therapeutic for HIE.

## 1. Introduction

Perinatal hypoxic-ischemic encephalopathy (HIE; also referred to as birth asphyxia) remains the leading cause of neonatal mortality and morbidity, with an incidence in developed nations of 2–6 and 7 in every 1000 live term (>36 weeks gestation) and pre-term (≤36 weeks gestation) births, respectively [1,2]. The incidence is even higher in developing countries, with rates of up to 30 per 1000 live full-term births [3,4]. Of the individuals affected by HIE, 15–20% will die in the postnatal period, and an additional 25% will develop severe and permanent neurological sequela. Each year, HIE accounts for up to 1.2 million deaths [5] and some form of central nervous system (CNS) dysfunction in an estimated 1.15 million neonates [6] such as cerebral palsy, epilepsy, global developmental delay, intellectual disability, behavioral disorders and autism spectrum disorders [7,8,9,10,11]. While hypothermia is used as a therapeutic intervention to minimize brain injury in neonates suffering HIE, it only provides beneficial outcomes in approximately 30% of patients and has several limitations (reviewed below). Therefore, an effective and easily administrable pharmacological therapeutic agent to improve patient outcomes when used as an adjunct to hypothermia, on its own or when hypothermia cannot be applied is urgently needed.

In recent years, there has been an increased application of putative neuroprotective peptides designed to target cyto-damaging or cyto-protective pathways to reduce injury in acute brain disorders such as HIE. One reason for this interest is the discovery of cell-penetrating peptides (CPPs), allowing for the delivery of neuroprotective peptides and other molecular cargos (e.g., proteins, nucleic acids and nanoparticles) across the blood brain barrier (BBB) and into cells within the CNS. Most studies assessing neuroprotective peptides in animal models of HIE use the cationic arginine-rich CPP, transactivator of transcription (TAT_47–57_ YGRKKRRQRRR), as the ‘carrier’ molecule. However, several years ago, our and other laboratories demonstrated that the TAT peptide has modest intrinsic neuroprotective properties in its own right [12,13,14,15,16,17]. We subsequently demonstrated that other cationic arginine-rich peptides [CARPs; e.g., penetratin, protamine, sodium calcium exchanger inhibitory peptide (XIP), poly-arginine peptides (e.g., R9, R12 and R18; R indicates amino acid arginine and number indicates mer)], possess potent neuroprotective properties in both in vitro and animal models of cerebral ischemia and/or hypoxia [12,18,19,20,21,22,23,24,25,26,27,28,29], and demonstrated that the arginine content and peptide positive charge are critical determinants of neuroprotection [19,27,28].

Based on the recent application of cationic CPPs for the delivery of putative neuroprotective peptides in animal models of HIE and the recent discovery of the intrinsic neuroprotective properties of CARPs, the objectives of this review are: (i) catalogue studies that have used peptides as neuroprotective agents in HIE, especially those employing a CPP delivery system; and (ii) to evaluate the potential neuroprotective effects of both CPP and cargo molecules in terms of their arginine content and cationic charge, as well as the content of other potentially neuroprotective amino acids such as tryptophan [29]. We will propose that the neuroprotective properties of most, if not all putative neuroprotective peptides fused to cationic CPPs are likely to be mediated in a large part by the actions of the carrier CPP (e.g., TAT) rather than the cargo molecule itself, with potency being further enhanced according to the amino acid content of the cargo (e.g., number of arginine residues) [19]. In addition, we will also review the evidence that CARPs have multiple cellular modes of action that underlie their neuroprotective properties.

First, we provide a brief overview of the major underlying pathophysiological mechanisms associated with HIE and the application of hypothermia as an acute therapy for the disorder.

## 2. Pathophysiology of Perinatal Hypoxia-Ischemic Brain Injury

### 2.1. Initiation of the Pathophysiological Cascade in HIE

The etiology of HIE is associated with a variety of maternal, placental and fetal conditions; all of which have variable clinical manifestations. These conditions include, but are not limited to, chronic maternal hypoxia (e.g., chronic utero-placental hypoxia, cardiopulmonary arrest, acute hypotension, pulmonary embolism or vascular disease), pre-eclampsia, nuchal cord, umbilical cord knotting, umbilical cord prolapse, shoulder dystocia and premature placental detachment [30]. Perinatal hypoxia is initiated following an impairment of oxygenated cerebral blood flow (CBF) to the fetus, triggering responses at both the systemic and cellular levels. The fetal brain is particularly sensitive to a HI environment, as there is a requirement of a constant supply of energy in the form of ATP, and when interrupted, excitotoxicity occurs through the uncontrolled release of excitatory neurotransmitters such as glutamate; marking the beginning of the ischemic cascade. The acute and downstream consequences of the ischemic cascade are damaging to neurons and other cells (e.g., glial progenitor cells and astrocytes) at the cytoplasmic and mitochondrial levels, as well as causing disruption to the BBB and the activation of inflammatory responses.

### 2.2. Excitotoxicity

The metabolic rate of the fetal brain is largely determined by the stage of gestational development [30]. To meet metabolic demands, developing neural tissue metabolizes lactate, ketone bodies and glucose. When compared to the developed brain, the fetal brain is more adaptable with respect to energy utilization, thereby increasing its capacity to tolerate hypoxia-ischemia (HI) [31]. However, in the event of a critical depletion of ATP due to prolonged HI, the fetal brain eventually becomes susceptible to injury.

Following prolonged HI, cellular homeostasis is disrupted due to the depletion of ATP and failure to maintain ionic gradients, and as a result, neurons depolarize and release glutamate into the synaptic cleft [32]. High extracellular levels of glutamate cause excitotoxicity in neurons and other cells, namely glial progenitor cells, that express glutamate receptors (NMDA; *N*-methyl-d-asparate, AMPA; α-amino-3-hydroxy-5-methy-4-isoxazolepropionic acid, kainic acid and metabotropic glutamate receptors), producing a cytotoxic cellular influx of calcium ions [33]. The increased levels of intracellular calcium in neurons and glial progenitor cells in turn results in the activation of calcium-dependent proteases, lipases and deoxyribonuclease (DNase), reactive oxygen species (ROS) production, oxidative stress, cytotoxic edema, mitochondrial dysfunction and the stimulation of pro-cell death pathways [34,35,36,37,38].

### 2.3. Oxidative Stress

Oxidative stress plays an important role in the pathophysiology of HIE, as the developing brain comprises high levels of unsaturated fatty acids susceptible to lipid peroxidation, metals catalyzing free radical reactions and low concentrations of antioxidants [39]. The resulting heightened sensitivity to oxidative stress and ROS production following HI causes significant damage to lipids (lipid oxidation), nucleic acids (DNA degeneration) and proteins (protein oxidation). Sources of ROS following HI include, the mitochondrial electron transport chain (ETC), NADPH oxidases (NOX), xanthine oxidase (XO), arachidonic acid (12/15 lipoxygenase), and nitric oxide synthase (NOS). In particular, NOS has been targeted in HIE, with strategies to ablate or inhibit NOS activity invariably being neuroprotective in animal models of HIE [40].

### 2.4. Mitochondrial Dysfunction

Mitochondria have diverse functions, including ATP generation, intracellular calcium regulation, ROS production, biosynthesis of amino acids, lipids and nucleotides and pro-cell death signaling. The role of mitochondria in HIE has been extensively reviewed [41,42,43]. Here we focus on the role of mitochondria in potentiating secondary pathophysiological cascades contributing to HI. Mitochondria contribute to secondary brain injury following HI via the regulation of cell death pathways, namely apoptosis. Cellular apoptosis following HI is activated via both intrinsic (e.g., in response to mitochondrial dysfunction and damaged DNA) and extrinsic [e.g., in response to cell death receptors such as Fas and tissue necrosis factor-α (TNF-α)] pathways and involve the release of pro-cell death proteins from mitochondria.

In cells affected by HI, several processes such as excitotoxicity, ROS production, activation of p53, DNA damage and altered mitochondrial function can influence the activity of pro-apoptotic Bcl-2 proteins (e.g., Bid and Bax), resulting in Bax-dependent mitochondrial membrane permeability transition (MPT) [44,45]. In addition, excessive mitochondrial ROS generation, due to leakage from the ETC, can oxidize the inner mitochondrial membrane phospholipid, cardiolipin, promoting mitochondrial membrane permeability transition pore (MPTP) opening and subsequent release of cytochrome-c. Cytosolic cytochrome-c, along with deoxyadenosine triphosphate (dATP), interact with apoptotic protease activating factor-1 (Apaf-1) to form the apoptosome, which cleaves and activates pro-caspase-9 [46]. Activated caspase-9 in turn activates caspase-3, which is a major proteolytic enzyme responsible for the ultimate dismantling of cells during apoptotic cell death.

Further alterations to mitochondria, such as mitochondrial outer membrane permeabilization (MOMP), results in the release other pro-apoptotic factors into the cytosol, including apoptosis-inducing factor (AIF), endonuclease G (Endo-G), and Smac/DIABLO [47,48]. Translocation of AIF and Endo-G to the nucleus mediates chromatin fragmentation and Smac/DIABLO interacts with inhibitors of apoptosis to reduce their actions on activated caspases.

TNF cell death receptor signaling mainly stimulates the extrinsic pathway by activating caspase-8, which activates other caspases, including caspase-3. While beyond the scope of this review, it is also important to note that other cell death pathways associated with necrosis, necroptosis, ferroptosis and autophagy may also be activated in response to HI. These pathways can be activated in response to one or more cellular disturbances such as calcium influx, mitochondrial dysfunction, cellular energy depletion, ROS production, TNF receptors cell signaling and other cellular cell signaling pathways (e.g., adenosine monophosphate-activated protein kinase).

### 2.5. Inflammation

The inflammatory response following HIE has been extensively reviewed by [49,50] and is therefore briefly discussed. In the immature brain following HI, an innate immune response occurs within minutes of the injury [51]. Initially, microglia become activated within the cerebral parenchyma, followed by a systemic inflammatory response, mediated by the infiltration of circulating monocytes, neutrophils and T-cells into the brain. Activated microglia develop macrophage-like attributes, such as phagocytic properties, cytokine production, antigen presentation and matrix metalloproteinase (MMP) release. The release and activation of MMPs by microglia can compromise BBB integrity, further promoting the invasion of peripheral leukocytes into the cerebral parenchyma; exacerbating ensuing cerebral injury. A hallmark of HIE is the aggregation of amoeboid microglia within the periventricular white matter, resulting in the production of inflammatory cytokines (TNF-α and IL1-β), NO and other ROS [52]; collectively potentiating the toxic effects of the ischemic cascade in neurons, glial progenitor cells and the cerebral vasculature.

While astrocytes attempt to provide neuroprotective support to neurons and glia via the release of glutathione, superoxide dismutase (SOD), extra-synaptic uptake of glutamate and the maintenance of ion channel gradients, these protective mechanisms can be quickly overrun. As with microglia, astrocytes become hyper-activated due to the effects of pro-inflammatory cytokines, ROS, and dying neurons and glia. Activated astrocytes also release pro-inflammatory cytokines (IL-6, TNF-α, IL-1α/β and INF-γ), further exacerbating HI-induced cell death pathways (e.g., apoptosis). In addition, astrocytes secrete chemokines that attracts systemic immune cells to infiltrate affected tissue, further aggravating tissue injury.

## 3. Current Clinical Treatments: Hypothermia

Hypothermia is considered one of the most effective neuroprotective treatment interventions in both experimental and clinical settings. While the exact beneficial effects of hypothermia are yet to be elucidated in HIE, it is known to suppress many of the neuro-damaging events associated with HI including excitotoxicity, apoptosis, inflammation, oxidative stress and BBB disruption [53].

In HIE, moderate hypothermia (33.5 °C for 72 h), applied within 6 h of birth, improves the survival and neurodevelopmental outcomes in infants suffering from moderate and severe HIE. While hypothermia is now considered standard care in the treatment of HIE and is well tolerated in term infants [54,55,56,57], it does not provide complete protection. Of the infants who have moderate or severe encephalopathy, therapeutic hypothermia was shown to decrease mortality from 40% to 28%, while in surviving infants neurological morbidity was reduced from 31% to 19% of infants receiving therapeutic hypothermia [58].

Importantly, while hypothermia is safe in pre-term infants with necrotising enterocolitis [59], it has not been adequately evaluated for use in pre-term infants suffering HIE. Information regarding the safety and efficacy of hypothermia to treat pre-term infants suffering HIE is lacking, although information may be available in the near future (NCT1793129). In one study, hypothermia in pre-term infants was associated with higher mortality and increased clinical complications when compared to term infants [60], although the study did not include a normothermic pre-term control group. The inadequate application of hypothermia in the pre-term neonate highlights the need for the development of safer therapeutic interventions in these patients following HI.

In addition, the induction of hypothermia requires specialized equipment, intensive care monitoring and trained staff, limiting its use to centers that have the facilities and medical personnel to undertake the procedure. This develops uncertainty regarding the timing, safety and efficacy of initiating hypothermia in infants before transfer to a centre equipped to deliver hypothermia. For example, the transfer of infants suffering HIE to a medical centre to provide hypothermia can exceed 6 h from the time of birth and that attempts of passive cooling during transport can result in excessive cooling [61]. In light of this, there is an urgent need for the application of an acute neuroprotective therapy for infants who suffer HIE when hypothermia cannot be applied immediately, or is not available. In addition, as the incidences of mortality and morbidity are still relatively high following hypothermia, there is a requirement for additional therapeutic agents, adjuvant to hypothermia, to further improve outcomes in infants suffering HIE.

## 4. Neuroprotective Peptides and Their Therapeutic Application in HIE

### 4.1. Peptide Therapeutics

Peptides represent an important and increasingly popular class of therapeutic agent. To date, over 60 general peptide drugs have been approved in the United States, Europe, and Japan, approximately 140 are in active clinical development and more than 500 peptides are being examined in preclinical therapeutic studies [62]. Peptides have several beneficial properties over traditional drugs such as high biological and structural diversity, less toxicity and accumulation in tissue and reduced propensity to cause drug-drug interactions. The main disadvantage of most peptides is their inability to cross the BBB and enter cells, which is an important issue if the peptide has an intracellular target and/or is targeting a CNS disorder. However, as outlined above, the delivery of peptides into the CNS and into cells can be achieved by using CPPs. For this reason, CPPs have been widely utilized as a carrier molecule for the delivery of neuroactive compounds into the CNS, including putative neuroprotective peptides. Below we review experimental studies that have used neuroprotective peptides in HIE models, including putative neuroprotective peptides fused to a CPP. First, we summarize findings from our own and other laboratories highlighting the intrinsic neuroprotective properties of CARPs.

### 4.2. CARPs Are Intrinsically Neuroprotective

An early study by Ferrer-Montiel et al. [63], demonstrated that cationic arginine-rich hexapeptides (i.e. containing 2–6 arginine residues; net charge +3–+7; e.g., RRRRRR-NH_2_) were neuroprotective in an in vitro hippocampal neuronal NMDA excitotoxic model. These findings were in line with subsequent studies in our and other laboratories, demonstrating that the CPP TAT (YGRKKRRQRRR; net charge +8) possesses intrinsic neuroprotective properties following in vitro glutamic acid excitotoxicity and oxygen glucose deprivation (OGD), as well as in vivo following excitotoxicity and cerebral ischemia in rats [13,14,15,16,64,65]. We subsequently demonstrated that in an in vitro cortical neuronal excitotoxic model, the CPPs poly-arginine-9 (RRRRRRRRR-NH_2_; net charge +10) and penetratin (RQIKIWFQNRRMKWKK-NH_2_; net charge +8) were even more potent than TAT [12]. The high potency of R9 led us to explore other poly-arginine peptides (e.g., R1, R3, R6–R15, R18, R22), as well as several other CARPs, including protamine in the in vitro excitotoxic and OGD neuronal injury models [27,28,29]. These studies confirmed that CARPs are highly neuroprotective, with efficacy increasing with arginine content and peptide positive charge; peaking at R15 to R18 for poly-arginine peptides [27,29] [Note arginine and lysine (K) are the only positively charged amino acids, while histidine has a low positive charge].

Our laboratory has also demonstrated that CARPs and in particular the poly-arginine peptide R18 are neuroprotective in in vivo experimental brain ischemic and/or hypoxic injury models [18,20,21,22,24,26,27,29], and that CARPs can reduce neuronal cell surface glutamate receptor levels and excitotoxic calcium influx [19,22,23,25,27,28]. As highlighted previously [19], based on our findings, we have proposed that the neuroprotective properties of most, if not all, putative neuroprotective peptides fused to cationic CPPs (e.g., TAT, R9 and penetratin), as well as other CARPs, are determined by the arginine content and positive charge of the peptide, with potency also influenced by other amino acid residues (e.g., lysine, tryptophan and phenylalanine) [19,29]. Importantly, an arginine and cationic charge mediated neuroprotective mechanism of action of CARPs, including CPP-fused to putative neuroprotective peptides (e.g., TAT-NR2B9c and TAT-NR2Bct), is supported in studies by Marshall et al. [65] and more recently McQueen et al. [66]. Based on studies from our own and other laboratories, we believe that the mechanism of action of putative neuroprotective peptides fused to a CPP need to be critically re-evaluated. In light of the potentially confounding effects of CPPs and/or peptide arginine content and positive charge, we will review studies using neuroprotective peptides in HIE models. First, we provide a summary of the known and potential neuroprotective actions of CARPs.

### 4.3. Cationic Arginine-Rich Cell-Penetrating Peptide Neuroprotective Mechanisms of Actions

While the exact mechanisms of the neuroprotective effects are still being explored, we and others have demonstrated that CARPs have multiple, potentially simultaneous mechanisms of action. CARPs and putative neuroprotective peptides fused to cationic arginine-rich CPPs (i.e., TAT or R9; e.g., JNKI-1-TATD, TAT-NR2B9c/NA-1, TAT-CBD3/R9-CBD3-A6K and TAT-3.2-III-IV) can mitigate excitotoxic or potassium evoked neuronal intracellular calcium influx [19,27,67,68,69]. One mechanism whereby CARPs have the capacity to reduce intracellular calcium influx is the reduction of neuronal cell surface ion channels and receptors; thereby reducing excitotoxic calcium influx. To this end, CARPs have been demonstrated to reduce the cell surface expression or function of neuronal NMDA receptors [23,63,70,71,72,73], voltage gated calcium channels (CaV2.2 and CaV3.3) [68,70,74,75], a voltage gated sodium channel (NaV1.7) [76] and the sodium calcium exchanger 3 (NCX3) [70]. Given their effects on different receptors, and the positive charge of CARPs, they may also be antagonizing ion channel receptor function directly and/or by affecting calcium or sodium ion influx via an electrostatic mechanism.

In addition to neuronal cell surface receptor interactions, CARPs may also exert pleiotropic neuroprotective effects by targeting mitochondria and maintaining mitochondrial integrity by reducing calcium influx into the organelle [65,77,78,79,80,81,82,83]. CARPs have the capacity to inhibit proteolytic enzymes including the proteasome [84,85], as well as pro-protein convertases [86,87,88] that activate MMPs [89]. Inhibition of the proteasome is known to be beneficial following cerebral ischemia [90,91,92]; a mechanism that could be mediated by increased activity of hypoxia-inducible factor 1-α (HIF1α) and decreased activity of nuclear factor κ-light-chain-enhancer of B cells (NFκB). Similarly, MMP activation in the brain is known to be detrimental following cerebral ischemia and inhibiting MMP activation is neuroprotective (see review Cunningham et al. [93]).

Furthermore, there is also evidence that CARPs activate pro-survival signaling pathways [67,94], modulate immune responses [95,96,97] and act as anti-oxidant molecules in their own right due to their guanidine group in arginine residues [65,83,98,99,100,101].

Interestingly, the neuroprotective potency of CARPs appears to correlate with the same characteristic that improve the ability of cationic CPPs to target and transverse cellular membranes [19,29]. This suggests that CARP-mediated neuroprotection is associated with cellular membrane interactions (e.g., plasma and mitochondrial), anionic structures (phospholipids/cardiolipin, heparan sulfate proteoglycans, chondroitin sulfate proteoglycans, sialic acid resides, anionic regions of surface receptors) and/or increased levels of cellular uptake, which enable the peptides to interact with membrane (e.g., ion channel receptors) and intracellular targets (e.g., proteins, ROS and mitochondria).

### 4.4. Studies Using CARPs in Animal Models of HIE

All descriptive details of the studies discussed below are presented in chronological order of publication (Table 1).

#### 4.4.1. TAT-NEMO Binding Domain (NBD)

NEMO binding domain (NBD) is an 11-amino acid peptide (NBD: TALDWSWLQTE_735–745;_ net charge −2) derived from the Nuclear Factor-κβ (NF-κβ) Essential Modulator (NEMO)/IKK-γ-Binding Domain. The NBD peptide was designed to inhibit the activation of NFκB, a transcription factor involved in many stress responses. For example, NBD was originally developed to suppress inflammatory responses (e.g., IL-6, iNOS, MMP-9 and COX-2) and the regulation of apoptotic Bcl proteins (e.g., Bcl-2 and Bcl-x) following cytokine stimulation in several disorders including inflammatory bowel disease, arthritis, type I diabetes, multiple sclerosis and Parkinson’s disease [102]. Activation of NF-κB requires upstream kinase complex formation (IκB-kinase; IKK) composed of the IKKα, IKKβ and NEMO proteins. For NF-κB activation, NEMO interacts with a carboxyl terminal sequence within both IKK-α and IKK-β, known as the NBD. The NBD peptide inhibits NF-κB activation by blocking NEMO/IKK complex formation [103].

The first study examining the neuroprotective efficacy of the NBD peptide in a model of HIE (P12 rat) administered the peptide [(14,820 nmol/kg: intraperitoneally (IP)] immediately, 6 and 12 h after hypoxia and did not demonstrate any neuroprotection; instead NBD appeared to increase brain injury [104]. To improve delivery to the brain, the NBD peptide was fused to TAT (TAT-NBD: YGRKKRRQRRR-TALDWSWLQTE; net charge +6) and assessed in a P7 rat model of HIE [105], where administration (6917 nmol/kg: IP) immediately and 3 h, or as a single injection at 0, 3 or 6 h, after hypoxia was neuroprotective up to 6 weeks post-HI. When TAT-NBD was administered immediately and 3 h after hypoxia it prevented up-regulation of p53 and activation of caspase-3 24 h post-HI. TAT-NBD treatment also prevented the activation of IKK in the brain 3 h post-HI. Despite TAT-NBD inhibiting IKK and NF-κB activation, it had no effect on HI-induced increases in cytokines (e.g., IL-1β, TNF-α, IL-4, IL-10 and IL-1-RA). The study also assessed a TAT-NBD control peptide, where two tryptophan residues were substituted for two alanine residues (YGRKKKRRQRRR-TALD**A**S**A**LQTE; net charge +6) and was shown not to be neuroprotective. Further assessment of TAT-NBD (6817 nmol/kg: IP) involving administration immediately and 3 h, immediately, 3, 6 and 12 h, immediately, 6 and 12 h, as well as 18 and 21 h after hypoxia [106], revealed long-term neuroprotection only in animals treated immediately and 3 h post-HI. Concomitantly, TAT-NBD treatment immediately and 3 h after hypoxia significantly reduced HI-mediated cytokine levels, whereas immediate, 3, 6 and 12-h treatment did not reduce cytokines. In a subsequent study, TAT-NBD treatment (6818 nmol/kg: IP) immediately and 3 h after hypoxia demonstrated significant improvements in behavioral outcomes up to 14 weeks post-HI [107].

Since intrauterine infections can be associated with HIE, TAT-NBD was assessed in a lipopolysaccharide (LPS)-sensitized (IP injection) HIE model in the P7 rat [108]. Animals were administered with TAT-NBD (477 nmol/kg) intranasally (IN) 10 min after hypoxia, using three HI injury conditions; HI only, 4-h LPS-pre-treatment + HI or 72-h LPS-pre-treatment + HI. Treatment with TAT-NBD significantly reduced brain injury only in the LPS-sensitized HI models, when assessed 7 days post-HI. While it was surprising that TAT-NBD did not reduce brain injury following HI only, its neuroprotective actions in LPS-sensitized HI models may be due to the ability of the cationic TAT-NBD peptide to electrostatically bind and neutralize anionic LPS molecules or by reducing the effects of inflammatory cytokines. Neutralizing LPS would have the effect of suppressing the molecules capacity to evoke an immune response and inflammatory response. In support of this mechanism, previous studies using in vitro cell and in vivo tissue injury models have demonstrated that CARPs (e.g., HBD-2, HNP-1 and indolicidin) can bind to LPS and reduce its inflammatory effects [109]. Furthermore, it is possible that the IN delivery of TAT-NBD was enough to reduce the detrimental effects of LPS, but not ischemic injury associated with HI.

#### 4.4.2. TAT-mGluR1

mGluR1 is a 14-amino acid peptide (mGluR1: VIKPLTKSYQGSGK_929–942_; net charge +3), corresponding to an intracellular region of the metabotropic glutamate receptor-1α (mGluR1α) protein that is cleaved by calpain in neurons following excitotoxicity [110]. It is proposed that TAT-mGluR1 (YGRKKRRQRRR-VIKPLTKSYQGSGK, net charge +11) blocks the ability of calpain to cleave mGluR1α and thus maintain the receptors ability to stimulate the neuroprotective phosphoinositide 3-kinase/protein kinase B (PI3K-Akt) signaling pathway and protect neurons from the toxic effects of excitotoxic calcium influx [110]. In a neuronal model of kainic acid mediated excitotoxicity, TAT-mGluR1 prevented mGluR1α truncation and reduced neuronal cell death [110]. In a P7 rat model of HIE [111] TAT-mGluR1 (58,590 nmol/kg; IP) administered 1 h before hypoxia significantly reduced cerebral infarction at 24 h post-HI.

#### 4.4.3. c-Jun N-Terminal Kinase (JNK) Inhibitors

JNKI-1 is a 20-amino acid peptide (also referred to as JBD: RPKRPTTLNLFPQVPRSQDT_157–176_; net charge +3), derived from the signaling adaptor protein c-Jun N-terminal kinase protein-1 (JNKI-1) [112]. JNKs are a member of the mitogen-activated protein kinases (MAPK) family, encoded by the *Jnk1*, *Jnk2* and *Jnk3* genes, with high expression levels in neurons, especially after pathological injury (e.g., excitotoxicity, stroke, epilepsy and HI) [113]. The JNKI-1 peptide can inhibit JNK interaction with JNK-interacting protein-1 (JIP-1), blocking JNK phosphorylation and activation, thereby inhibiting downstream pro-death cellular signaling pathways [112]. JNK has emerged as a central mediator of excitotoxic damage in the developing [114,115] and developed CNS [116,117]. The JNKI-1 peptide derivatives bound to TAT, such as TAT-JNKI-1L (YGRKKRRQRRR-PP-RPKRPTTLNLFPQVPRSQDT-NH_2_, net charge +12) and its retro inverso D-enantiomer JNKI-1-TATD (tdqsrpvqpflnlttprkpr-pp-rrrqrrkkrgy-NH_2_; net charge +12, lower case indicates D-isoform amino acids) have demonstrated in vitro and/or in vivo neuroprotective properties. Studies using the JNKI-1 peptide alone (D-JNKI-1) or when fused to TAT (TAT-JNKI-1L and JNKI-1-TATD) have been assessed in neonatal HIE models.

The initial study examining the efficacy of D-JNKI-1 (tdqsrpvqpflnlttprkpr-NH_2_; net charge +4) in a P7 rat model of HIE when administered (76 nmol/kg: IP) 30 min before and 3, 5, 8, 12 and 20 h after hypoxia did not reveal any reduction in cerebral infarction at 24 h [115], although there was evidence for reduced calpain, caspase-3 and autophagic activation.

A subsequent study demonstrated that the TAT-fused peptide JNKI-1-TATL administered (2446 nmol/kg; IP) immediately and 3 h or 3 h after hypoxia, significantly reduced cerebral infarction at 48 h, while administration 6 h after hypoxia was ineffective [118]. When administered immediately and 3 h after hypoxia, functional benefits were observed 14 weeks post-HI. Despite improvements in cerebral infarct and functional outcomes, JNKI-1-TATL failed to prevent caspase-8-mediated cleavage of Bid, which was unexpected, as activated JNK is known to induce caspase-8 cleavage of Bid and promote mitochondrial pro-apoptotic cell death pathways; this suggests JNKI-1-TATL-mediated neuroprotection was occurring via mechanism independent of JNK suppression.

In a second study, the _D_-isoform peptide JNKI-1-TATD (2616 nmol/kg: IP) significantly reduced cerebral infarct volume when administered immediately, 3 or 6 h after hypoxia, but not when administered immediately and 3 h after hypoxia [119]. JNKI-1-TATD treatment also provided long-term functional improvements. It was also demonstrated that treatment with JNKI-1-TATD reduced mitochondrial levels of phosphorylated JNK, preserved mitochondrial integrity, and up-regulated anti-apoptotic proteins 24 h post-HI. The study also assessed a mitochondrial JNK scaffold inhibiting peptide, Sab_KIM1_ (gfeslsvpspldlsgprvva-pp-rrrqrrkkrg; net charge +7) and a scrambled control (lpsvfgdvgapsrlpevsls-pp-rrrqrrkkrg; net charge +7); Sab (SH3 domain-binding protein 5) is a mitochondrial scaffold protein required for JNK kinase activity. Administration of Sab_KIM1_ (2700 and 5555 nmol/kg: IP) immediately after hypoxia was neuroprotective, whereas the scrambled peptide (2700 nmol/kg: IP) was ineffective.

In our laboratory, administration of JNKI-1-TATD (1000 nmol/kg; IP) immediately after hypoxia resulted in a positive trend in reduced total infarct volume (15% reduction) although it did not improve behavioral outcomes 48 h post-HI [22]. It was also demonstrated that in cultured cortical neurons JNKI-1-TATD dose-dependently reduced glutamic acid mediated excitotoxic intracellular calcium influx.

While it was surprising that the Sab_KIM1_ scrambled control peptide did not display any neuroprotection, it is possible that the amino acid sequence of the cargo peptide may have influenced neuroprotective potency. Importantly, we and others have confirmed that TAT-fused scrambled peptides such as TAT-p53DMs and TAT-NR2cts [29,66] still retain neuroprotective properties, again pointing to an active role of the TAT carrier and peptide arginine content mediated neuroprotective action.

#### 4.4.4. TAT-BH4

BH4 is a 25-amino acid peptide derived from the Bcl-x(L) protein BH4 homology domain region (RTGYDNREIVMKYIHYKLSQRGYEW_6–30_, net charge +2.1). The BH4 domain is required by Bcl-x(L) to block cytochrome-c release from mitochondria and prevent MPTP opening [120], as well as inhibiting endoplasmic reticulum (ER) calcium release into the cytosol by binding to a regulatory domain of the inositol triphosphate-3 (IP3) receptor [121]; highlighting potential mechanisms whereby the BH4 peptide could exert a neuroprotective action after HI.

The TAT-BH4 (YGRKKRRQRRR-RTGYDNREIVMKYIHYKLSQRGYEW, net charge +10.1) peptide was demonstrated to reduce cerebral tissue damage and caspase-3 activation when administered intracerebroventricularlly (ICV; 5 µL/20 ng) 30 min before hypoxia in a P7 rat model of HI [122]. Interestingly, exposure of cultured adult neural stem cells to TAT-BH4 promoted axonal remodeling, suggesting the peptide exerts other effects that may be beneficial in improving recovery following HI.

#### 4.4.5. Osteopontin (OPN) and TAT-Fused OPN Peptide (TAT-OPN)

Osteopontin (OPN) is a secreted acidic glycoprotein containing an arginine-glycine-aspartate (RGD) motif and possesses multiple cellular functions including chemotactic and cytokine-like actions. The OPN protein is expressed in various tissues including bone, placenta, pancreas and the CNS [123]. Following cerebral ischemia, OPN deficient mice demonstrate increased neurodegeneration [124] and when administered ICV, the protein is neuroprotective in several adult rodent cerebral ischemia and hemorrhagic stroke models [125,126,127,128].

Mechanisms thought to be associated with OPN-mediated neuroprotection include binding to the cell surface αvβ3 integrin receptor to stimulate pro-survival signaling (i.e., PI3K/Akt pathway), anti-inflammatory effects [126,129,130,131], inhibition of inducible NOS [132,133] STAT1 ubiquitination/degradation and by reducing MMP-9 and NF-κβ activation. Despite OPN’s demonstrated neuroprotective effects, in one study OPN deficient mice demonstrated no change in susceptibility to ischemic brain injury [126].

The first study to examine the neuroprotective efficacy of OPN in HIE used full length recombinant OPN protein (rOPN) in a P7 rat model of HI [134]. Animals treated with rOPN (0.03 or 0.1 µg per animal: ICV) 60 min after hypoxia, displayed a reduction in infarct volume at 48 h and significant improvements in behavioral outcomes at 7 weeks post-HI. In contrast, treatment with rOPN (0.05 µg/animal: ICV or 1.2 µg/animal: IN) or thrombin cleaved rOPN (1.2 µg/animal: IN) was not neuroprotective in a P5 mouse model of HI [135]. To improve cerebral delivery of OPN, a shorter peptide containing the RGD motif (IVPTVDVPNGRGDSLAYGLR_134–153_; net charge 0) was developed. However, the shorter peptide was not neuroprotective when administered ICV (0.2 µg/animal) or IN (30 µg/animal) before hypoxia in P5 mice [135]. In a subsequent study the OPN_134–153_ peptide was fused to TAT (TAT-OPN; YGRKKRRQRRR-IVPTVDVPNGRGDSLAYGLR, net charge +8) and assessed in a P9 mouse model of HI [136] using several peptide treatment regimens consisting of multiple dosing using IN, IP or ICV administration (see Table 1 for details). No treatment regimen reduced infarct volume or improved functional assessments up to 35 days post-HI. Taken together, current evidence indicates that at the dosing schedules examined for the TAT-OPN peptide, it is not neuroprotective in HIE rodent models.

#### 4.4.6. P5-TAT

P5 is a 24-amino acid peptide derived from the C-terminal region of the p35 protein (KEAFWDRCLSVINLMSSKMLQINA_254–277_, net charge +0.9). Following cleavage of p35 by calpain, a cleavage fragment known as p25 can bind to cyclin-dependent kinase 5 (Cdk5) and alter its cellular location and stimulate its activity [137], which is known to be associated with neuronal cell death [138,139,140]. Administration (3,660, 7,320 and 9,761 nmol/kg: IP) of a modified P5 peptide (TFP5: KEAFWDRCLSVINLMSSKMLQINAYARAARRAARR; net charge +5.9) can improve outcomes in Parkinson’s disease, Alzheimer’s disease, and adult ischemic stroke models in rodents [140,141,142].

A dose-response assessment (0.23, 2.30, 5.76 and 11.5 nmol/kg; IP) of P5-TAT (KEAFWDRCLSVINLMSSKMLQINA-YGRKKRRQRRR, net charge +8.9) when administered 30 min before hypoxia in P7 rats demonstrated that all doses, except 0.23 nmol/kg, significantly reduced cerebral infarct volume at 48 h [143]. In addition, P5-TAT, when administered (11.5 nmol/kg; IP) 24 h after hypoxia, significantly improved behavioral outcomes up to 3 weeks post-HI. However, P5-TAT treatment following HI did not have any effect on the levels of Cdk5 activators, p35, p25 or p29, despite suppressing cleavage of caspase-3.

#### 4.4.7. D-TAT-GESV

GESV is a 4-amino acid peptide (GESV, net charge −1) derived from the carboxyterminal PDZ motif (PDZ-G(D/E)XV; net charge −1) of nNOS. Proteins containing PDZ domains play a key role in anchoring receptor proteins in the membrane to intracellular cytoskeletal structures (e.g., actin and microtubules) and cytosolic proteins. Following NMDA receptor stimulation, nitric oxide synthase-1 adaptor protein (NOS1AP) via its PDZ binding domain, binds nNOS and recruits the protein to the cell membrane where it generates nitric oxide (NO). The D-TAT-GESV peptide (ygrkkrrqrrr-GESV, net charge +7) inhibits NOS1AP/nNOS binding, and is believed to confer neuroprotection by reducing NO production and activation of the p38 MAPK stress pathway [144]. In a P7 rat model of HIE, D-TAT-GESV (100 ng: ICV), administered before hypoxia reduced cerebral infarction at 7 days post-HI [145]. In comparison, the control peptide D-TAT-GASA (100 ng: ICV) was ineffective [145], which is not surprising since alanine residues can reduce CARP neuroprotective efficacy [19].

#### 4.4.8. TAT-NR2B9c/NA-1

The development of NR2B9c as a neuroprotective agent has recently been discussed in detail [146]. Briefly, NR2B9c is a 9 amino acid peptide (KLSSIESDV_1479–1487_) derived from the intracellular terminal carboxyl region of the NMDA receptor NR2B subunit protein, a site that binds the adaptor protein postsynaptic density-95 (PSD-95). PSD-95 couples nNOS to the NR2B subunit and following NMDA receptor activation stimulates NO generation. The NR2B9c peptide was developed on the basis that it blocks NR2B subunit and PSD-95 binding and thereby inhibiting NO production following NMDA receptor stimulation.

TAT-NR2B9c (YGRKKRRQRRR-KLSSIESDV; net charge +7) administered (19,850 nmol/kg; IP) either 110 min or 20 min before hypoxia in mice reduced brain injury at 24 h post-HI [147]. In addition, TAT-NR2B9c treatment significantly improved behavioral outcomes up to 7 days after HI. Interestingly, a 3000 nmol/kg dose (IP), administered 20 min before hypoxia was ineffective.

#### 4.4.9. Poly-Arginine-18 (R18 and R18D)

R18 (RRRRRRRRRRRRRRRRRR; net charge +18) and its D-enantiomer (rrrrrrrrrrrrrrrrrr; net charge +18) is an 18-amino acid peptide and has been demonstrated to have potent neuroprotective properties in various in vitro and in vivo stroke related injury models [18,24,26,29]. Moreover, we have recently demonstrated for the first time that R18 and R18D (30, 100, 300 and 1000 nmol/kg; IP), administered immediately after hypoxia, reduced cerebral infarct volume and improved behavioral outcomes at 48 h post-HI [22]. In an additional study, administration of R18D 30 (10, 30, 300 and 1000 nmol/kg; IP) and 60 min (30 nmol/kg; IP) after hypoxia, reduced cerebral infarct volume and improved behavioral outcomes at 48 h post-HI [21].

### 4.5. Other Peptides Examined in Animal Models of HIE

Several other peptides, not fused to a CPP, have also been examined in animal models of HIE. All descriptive details of the studies discussed below are presented in chronological order of publication and in Table 1.

#### 4.5.1. COG133

COG133 is a 20-amino acid peptide (TEELRVRLASHLRKLRKRLL_133–152_, net charge +5.1) derived from the apolipoprotein E (apoE) protein. COG133 has been demonstrated to be neuroprotective in several acute brain injury models and demonstrated to possess anti-oxidant, anti-excitotoxic and anti-inflammatory properties [148,149,150,151,152]. Exactly how the COG133 peptide imparts its anti-oxidant, anti-excitotoxic and anti-inflammatory properties is not known, but the positive charge of the peptide has been considered as a critical factor [19,148]. When examined in a P7 rat model of HIE, COG133 administration (40, 200, 300, 400 and 2000 nmol/kg: ICV) immediately before hypoxia displayed a neuroprotective dose-response effect [151].

#### 4.5.2. Connexin 43 (Cx43) Derived Peptides 

Astrocytic gap junction hemichannels play an essential role in the regulation of electrolytes and metabolites between the cytosol and extracellular space. Under physiological conditions, hemichannels are usually in a closed state. Specifically, connexin 43 (Cx43) hemichannel opening occurs following hypoxia, ischemia and OGD [153,154,155,156], resulting in a disruption to cellular homeostasis and an exacerbation of excitotoxicity [157]. The non-specific blocking of gap junction hemichannels with octanol and carbenoxolone demonstrates neuroprotective effects in in vitro models of hippocampal hypoxic-hypoglycemic injury [158,159]. To overcome the non-specific blocking of gap junction hemichannels, small mimetic peptides that bind to the extracellular loop of Cx43 hemichannels, blocking hemichannel opening have been developed [160].

Several studies have assessed the administration of a Cx43 mimetic peptide (Peptide 5: VDKFLSRPTEKT, net charge +1) in fetal sheep models of HIE. Administration of Peptide 5 continuously for either 1 (50,000 nmol/kg/h; ICV) or 25 h (50,000 nmol/kg over 1 h and then over 24 h; ICV), beginning 90 min after hypoxia, improved electroencephalographic (EEG) recovery, reduced seizure activity and improved the return of sleep state cycling. In addition, the 25-h infusion of Peptide 5 improved oligodendrocyte survival at 7 days post-HI [161]. Another study demonstrated Peptide 5 administration immediately after hypoxia (50,000 nmol/kg over 1 h and then over 24 h; ICV) improved neuronal and oligodendrocyte cell survival, whereas peptide administration beginning during hypoxia (50,000 nmol/kg for 1 h and then for 24 h; ICV) was not neuroprotective [162]. In contrast, a continuous high dose infusion of Peptide 5 (50,000 nmol/kg/h over 25 h: ICV) immediately after hypoxia did not improve neurological outcome and contributed to cerebral edema in the fetal sheep 7 days post-HI [163]. In a pre-term (103 days gestation) fetal sheep model of HI, Peptide 5 administration (50,000 nmol/kg for 1 h and then for 24 h; ICV) commencing 90 min after hypoxia improved EEG recovery, reduced neuronal death and increased oligodendrocyte cell survival 7 days post-HI [164]. A study using term fetal sheep assessed Peptide 5 and hypothermia (32 ± 1 °C; 72 h), as individual treatments and when combined. Peptide 5 administration 3 h after hypoxia (50,000 nmol/kg for 1 h and then for 24 h; ICV) and hypothermia treatments alone, improved EEG power, neuronal and oligodendrocyte cell survival at 7 days post-HI. However, combined Peptide 5 and hypothermia treatment did not result in any additional improvement in EEG power or histological outcomes [165]. This suggests that neuroprotective targets, other than hemichannels, may be more appropriate to improve the therapeutic effects of hypothermia for HIE.

Other Cx43 mimetic peptides (Gap 26: VCYDKSFPISHVR; net charge +1 and Gap 27: SRPTEKTIFII; net charge +1) have demonstrated neuroprotective efficacy in a P7 rat model of HIE [166]. Gap 26 (0.64, 3.22, 6.44, 16.1 and 32.2 nmol/kg; IP) and Gap 27 (16.1 nmol/kg; IP) administered 1 h before hypoxia, reduced infarct volume at 48 h post-HI; however, the two lower doses of Gap 26 were ineffective. In addition, multiple administrations of Gap 26 (32.2 nmol/kg: IP) every day for 7 days, beginning 24 h after hypoxia reduced brain injury and improved neurological function, up to 3 weeks post-HI.

Since Cx43 mimetic peptides Peptide 5, Gap26 and Gap 27 have only one arginine residue and low cationic charge, it would seem unlikely the peptides are operating via a CARP mechanism of action.

#### 4.5.3. Apelin-36

Apelin is an endogenous ligand of the apelin receptor, a G protein-coupled receptor, which is widely expressed throughout the body, including neurons and oligodendrocytes [167]. The human *APELIN* gene encodes a 77 amino acid pre-protein, that is cleaved into at least three biologically active fragments, Apelin-13_(65–77)_, Apelin-17_(61–77)_ and Apelin-36_(42–77)_. The active apelin fragments are widely expressed in the body including the CNS [168]. Apelin peptides have been demonstrated to reduce neuronal excitotoxicity and activate extracellular signal-regulated kinase 1/2 ERK-1/2 and PI3K/Akt cell survival pathways in cultured neurons [169,170,171]. Apelin-36 (LVQPRGSRNGPGPWQGGRRKFRRQRPRLSHKGPMPF_42–77_; net charge +10.1) has been assessed in a P7 rat model of HIE [172] with administration (1000 ng: IP) 1 h before hypoxia reduced infarct volume at 48 h post-HI, and improving neurological function up to 14 days post-HI.

#### 4.5.4. Innate Defense Regulator (IDR) Peptide IDR-1018

Innate defense regulator (IDR) peptides are endogenous cationic host defense peptides [173,174]. IDRs modulate inflammatory responses by increasing the recruitment of immune cells to sites of infection and inflammation; increasing wound-healing properties. It is believed that IDRs reduce cerebral injury following HI by reducing the impact of damaging inflammatory responses. IDR-1018 (VRLIVAVRIWRR-NH_2_; net charge +5) is a synthetic host defense peptide examined in an LPS-sensitized model of HIE in P9 mice [175]. IDR-1018 administered (5208 nmol/kg: IP) 4 h before hypoxia reduced brain injury-related gene pathways that include p53 activation and calcium signaling. In addition, IDR-1018 administered (5208 nmol/kg: IP) 3 h after hypoxia, reduced infarct and inflammatory mediators (e.g., TNF-α, IL-1β, IL-4, IL-6, IL-10, IL-17, keratinocyte chemoattractant, INF-γ and granulocyte-macrophage colony stimulating factor) 8 days post-HI.

## 5. Do All CARPs Including TAT-Fused Peptides Share a Common Neuroprotective Mechanism of Action?

Based on previous studies in our and other laboratories, examining the neuroprotective properties of various CARPs, we view it highly likely that the neuroprotection demonstrated by the CARPs described above (i.e., TAT-NBD, TAT-mGluR1, JNKI-1-TATL, JNKI-1-TATD, Sab_KIM1_, TAT-BH4, P5-TAT, D-TAT-GESV, TAT-NR2B9c, R18, R18D, COG133, Apelin-36 and IDR-1018) is largely mediated by peptide arginine content and positive charge [19,27,29,65,66]. There are several lines of evidence that support this hypothesis, which we provide below. In addition, we previously provided an explanation why we believe a CARP-mediated neuroprotective mechanism of action is operating for the TAT-NR2B9c and JNKI-1-TATD peptides, rather than the mechanism of action in which they were originally developed [27].

It has been demonstrated that CARPs inhibit neuronal excitotoxic calcium [19,22,23,25,27,28] and potassium [64,75] influx and in doing so inhibit many of the pathophysiological processes downstream from the ischemic excitotoxic cascade (e.g., calpain activation, mitochondrial dysfunction and ROS production, calcium induced cell signaling and lipase and DNase activation). Therefore, given that the vast majority of the proposed mechanisms of action of developed TAT-fused and other neuroprotective CARPs are molecular targets downstream from excitotoxicity (e.g., NF-κB, JNK and Cdk5), it is not surprising that they are effective and appear to be inhibiting their intended targets.

Interestingly, in addition to the inhibition of excitotoxic cationic ion influx, CARPs also have the capacity to modulate other cellular processes that are neuroprotective following HI such as proteolytic enzyme activation (e.g., proteasome and MMPs), ERK-1/2 and Akt phosphorylation and the modulation of immune responses. For example, in the case of TAT-NBD, which was developed to block NF-κB activation, the ability of CARPs to inhibit the proteasome [84,85,176,177] would have the effect of preventing NF-κB activation by preventing proteasomal degradation of the NF-κB inhibitor protein i-κB.

In the case of Apelin-36, other CARPs, such as protamine and R9 (ALX40-4C) can also bind to the apelin receptor [178,179]. Therefore, it is possible that CARPs, in addition to Apelin-36, may also be able to stimulate pro-cell survival ERK-1/2 and Akt pathways [180]. Although it should be mentioned that not surprisingly, Apelin-36 can also reduce excitotoxic calcium influx [169,170,171]. In the case of CARP-mediated immune modulation, mR18L (Ac-GFRRFLGSWARIYRAFVG-NH_2_; net charge +4) is known to reduce LPS-induced systemic infection [181], dRK6 (RRKRRR; net charge +6) inhibits TNFα and IL-6 production by monocytes [182] and hBD3-3 (GKCSTRGRKCCRRKK; net charge +8) downregulates NF-κB induced inflammation [183]. Thus, CARPs have the potential to suppress many of the toxic inflammatory responses associated with HIE.

Future studies examining the efficacy of putative neuroprotective peptides fused to TAT would benefit with respect to confirming neuroprotective mechanisms of action by fusing the peptide to a non-cationic CPP (e.g., MTS; AAVALLPAVLLALLP) and/or replacing any negatively charged aspartate (D) and glutamate (E) residues within the peptide sequence with arginine residues; we predict these two manipulations will decrease and increase neuroprotective efficacy, respectively. It is also advisable to confirm if the TAT-fused peptide has the capacity to reduce excitotoxic calcium influx.

## 6. Conclusions

There is now a large body of data from various sources demonstrating that CARPs comprise a class of peptide with significant neuroprotective potential for development as an adjuvant to hypothermia, or when hypothermia cannot be applied, for the treatment of HIE. While further studies are required to elucidate neuroprotective mechanisms, available evidence indicates that CARP neuroprotection is determined by the peptide’s arginine content and cationic charge and more specifically by the guanidine head group in the arginine residues. Interestingly, CARP neuroprotective potency appears to correlate with the same characteristics that improves the ability of the cationic CPPs to target and traverse cellular membranes [19,29]. Taken together it is clear that CARPs possess many properties enabling them to target both intracellular and extracellular damaging and protective pathways that are likely to be beneficial following HIE, not to mention their ability to cross the BBB and enter cells. In conclusion, CARPs, including poly-arginine peptides, offer an exciting therapeutic approach for HIE with the potential to reduce the severity of brain injury via potentially several neuroprotective mechanisms of action.

## 7. Future Directions

There are several further considerations to be addressed before translation of CARPs as a therapeutic for HIE in the clinic. It will be important to firstly identify a CARP that has high efficacy and the greatest potential to be effective in HIE, in both pre-term and term infants. In addition, preclinical studies will need to examine CARP treatment in combination with hypothermia to ensure safety, and to identify any synergistic effects in terms of improving patient outcomes. It is also important that beneficial results obtained in rodent models of HI, especially with combining CARPs and hypothermia, are confirmed in a large animal model (e.g., piglet or lamb), allowing for assessment in a gyrencephalic brain with greater comparability to the human and that the efficacy of IV administration should be established prior to clinical trials.

## Figures and Tables

**Table 1:** Studies using CARPs and other peptides examined in animal models of HIE.

Peptide/Protein Name; Net Charge ^a^	Proposed Target ^b^	Peptide Sequence ^c^	Injury Model ^d^	Route & Time before/after HI Agent Administered ^e^	Dose	NP ^f^	Study
**TAT-NBD; +6**	NFκB	TAT-TALDWSWLQTE	P7 (W): CCAO/8% O2; 120 min	IP: 0 & 3 h, or 0, 3, 6, 9 or 12 h	6917 nmol/kg	Yes, up to 6 h	[105]
				IP: 0 & 3 h, or 0, 9 & 12 h, or 18 & 21 h	6817 nmol/kg	Yes, only for 0 & 3 h	[106]
				IP: 0 & 3 h	6818 nmol.kg	Yes	[107]
			P7 (W): CCAO/10% O2; 90 min + LPS 4 or 72 h before hypoxia	IN; 10 min	477 nmol/kg	Yes, only for LPS	[108]
**TAT-mGluR1; +3**	Calpain	TAT-VIKPLTKSYQGSGK	P7 (SD): CCAO/8% O2; 150 min	IP: −1 h	58,590 nmol/kg	Yes	[111]
**D-JNKI-1; +4**	JIP	tdqsrpvqpflnlttprkpr-NH_2_	P7 (SD): CCAO/8% O2; 120 min	IP, −0.5, 3, 5, 8, 12 and 20 h	76 nmol/kg	No	[115]
**JNKI-1-TATL; +12**		TAT-PPRPKRPTTLNLFPQVPRSQDT-NH_2_	P7 (W): CCAO/8% O2; 120 min	IP: 0 and 3 h, or 3 h or 6 h	2446 nmol/kg	Yes, except 6 h	[118]
**JNKI-1-TATD; +12** **Sab_KIM1_ +7**		tdqsrpvqpflnlttprkpr-pp-tat-NH_2_ lpsvfgdvgapsrlpevsls-pp-tat	P7 (W): CCAO/8% O2; 120 min	IP: 0, 3 or 6 h, or 0 & 3 h IP: 0 h	2616 nmol/kg 2777 or 5555 nmol/kg	Yes except 0 & 3 h Yes	[119]
**JNKI-1-TATD; +12**		tdqsrpvqpflnlttprkpr-pp-tat-NH_2_	P7 (SD): ECA/CCAO/8% O2; 150 min	IP: 0 min	1000 nmol/kg	No	[22]
**TAT-BH4; +2.1**	Apoptosis	TAT-RTGYDNREIVMKYIHYKLSQRGYEW	P7 (SD♂): CCAO/8% O2; 150 min	ICV: −0.5 h	5 µL/20 ng	Yes	[122]
**T-OPN** **OPN_134–153_; 0** **OPN_154–198_; −4.9**	α_v_β_3_ integrin receptor	Thrombin cleaved OPN IVPTVDVPNGRGDSLAYR SKSRSFQVSDEQYPDATDEDLTSHMKSGESKESLDVIPVAQLLSM	P5 (C57B6/6J): CCAO/10% O2; 70 min	IN: −70 min IN or ICV: −70 min ICV: −70 min	1.2 µg IN: 30 µg/ICV: 0.2 µg 0.5 µg	No	[135]
**TAT-OPN; +8**		TAT-IVPTVDVPNGRGDSLAYGLR	P9 (C57BL/6N): CCAO/10% O2; 50 min	IN: 0 h or 0 & 3 h, or 0, 3 h, 1, 2 & 3 d IP: 0 h, or 5, 7, 9, 11, 13, 15 d, or 0, 1, 3, 5, 7, 9, 11, 13, 15 d ICV: 1 h	350 or 2100 ng 2746 nmol/kg 100 ng	No	[136]
**P5-TAT; +8.9**	P35	KEAFWDRCLSVINLMSSKMLQINA-TAT	P7 (SD): CCAO/8% O2; 150 min	IP: −1 h IP: 24 h	0.23, 2.3, 5.76, or 11.5 nmol/kg 11.5 nmol/kg	Yes, except 0.23 nmol/kg	[143]
**D-TAT-GESV; +7**	NOS	ygrkkrrqrrr-GESV	P7 (SD♂); CCAO/8% O2; 120 min	ICV: −120 min	100 ng/animal	Yes	[145]
**TAT-NR2B9c; +7**	PSD-95	TAT-KLSSIESDV	P7 (CD1): CCAO/7.5%; 60 min	IP: −110 or −20 min	3000 nmol/kg	Yes	[147]
**R18; +18** **R18D; +18**	Multiple	RRRRRRRRRRRRRRRRRR rrrrrrrrrrrrrrrrrr	P7 (SD): ECA/CCAO/8% O2; 150 min	IP: 0 h	30, 100, 300 or 1000 nmol/kg	Yes	[22]
**R18D; +18**		rrrrrrrrrrrrrrrrrr		IP: +0.5 h IP: +1 h IP: +1.5 h	10, 30, 100, 300 or 1000 nmol/kg 30 or 300 nmol/kg 30 or 300 nmol/kg	Yes, except 100 nmol/kg Yes, except 300 nmol/kg No	[21]
**COG133; 5.1**	LDLR	Ac-TEELRVRLASHLRKLRKRLL-NH_2_	P7 (W): CCAO/8% O2; 150 min	ICV: −0 h	40, 200, 300, 400, or 2000 nmol/kg	Yes, except 300 nmol/kg	[151]
**Peptide 5; +1**	Cx43 astrocytic hemichannel	VDKFLSRPTEKT	GD128 (Romney/Suffolk sheep): bilateral tCCAO; 30 min	ICV: 1.5 h	50,000 nmol/kg/h for 1 h ± 50,000 nmol/kg/24 h for 24 h	Yes	[161]
				ICV: −1 h or 0 h	50,000 nmol/kg/h: 1 h + 50,000 nmol/kg/24 h for 24 h	Yes, except −1 h	[162]
				ICV: 0 h	50,000 nmol/kg/h: 1 h + 50,000 nmol/kg/24 h for 24 h 50,000 nmol/kg/h for 25 h	Yes, except high continuous dose	[163]
				ICV: 3 h ± Hypothermia: 32 °C for 72 h; 3 h after tCCAO	50,000 nmol/kg/h for 1 h + 50,000 nmol/kg/24 h for 24 h	Yes, no additive effect	[165]
			GD103 (Romney/Suffolk sheep): bilateral tCCAO for 25 min	ICV: 1.5 h	50,000 nmol/kg/h for 1 h + 50,000 nmol/kg/24 h for 24 h	Yes	[164]
**Gap 26; +1** **Gap 27; +1**		VCYDKSFPISHVR SRPTEKTIFII	P7 (SD): CCAO/8% O2; 150 min	IP: −1 h IP: daily for 1 to 7 d IP: −1 h	0.64, 3.22, 6.44, 16.1, or 32.2 nmol/kg 32.2 nmol/kg 16.1 nmol/kg	Yes, except 0.64 and 3.22 nmol/kg Yes	[166]
**Apelin-36; +10.1**	Apelin receptor	LVQPRGSRNGPGPWQGGRRKFRRQRPRLSHKGPMPF	P7 (SD): CCAO8% O2; 150 min	IP: −1 h	240 nmol/kg	Yes	[172]
**IDR-1018; +5**	Immune modulation	VRLIVAVRIWRR-NH_2_	P9 (C57BL/6J): CCAO/10% O2; 20 min + LPS 14 h before hypoxia	IP: −4 h or 3 h	5208 nmol/kg	Yes	[175]

Bold data indicates peptides used in studies. ^a^ Net charge at pH 7, MUT: mutant peptide, SCR: scrambled peptide. ^b^ NFκB: nuclear factor kappa-light-chain-enhancer of activated B cells, JIP: c-Jun N-terminal kinase-interacting protein, NOS: nitric oxide synthase, PSD-95: postsynaptic density-95, LDLR: low-density lipoprotein receptor. ^c^ Peptides synthesized using d-amino acids are represented in lowercase, TAT = GRKKRRQRRR. ^d^ W: Wistar, SD: Sprague-Dawley, C57B/6: C57 black 6, CD1: cluster of differentiation 1, CCAO: common carotid artery occlusion, ECA: external carotid artery occlusion, tCCAO: transient common carotid artery occlusion, ♂ = male, GD = gestational day. ^e^ IP: intraperitoneal, IN: intranasal, ICV: intracerebroventricular, h: hours, min: minutes; negative (−) = treatment before hypoxia. ^f^ Neuroprotection and or positive effects.
